# Hepatitis B virus genotypes circulating in Brazil: molecular characterization of genotype F isolates

**DOI:** 10.1186/1471-2180-7-103

**Published:** 2007-11-23

**Authors:** Francisco CA Mello, Francisco JD Souto, Leticia C Nabuco, Cristiane A Villela-Nogueira, Henrique Sergio M Coelho, Helena Cristina F Franz, Joao Carlos P Saraiva, Helaine A Virgolino, Ana Rita C Motta-Castro, Mabel MM Melo, Regina MB Martins, Selma A Gomes

**Affiliations:** 1 Laboratório de Virologia Molecular, Instituto Oswaldo Cruz, FIOCRUZ, Rio de Janeiro, Brazil; 2 Faculdade de Ciências Médicas, Universidade Federal de Mato Grosso, Cuiabá, Brazil; 3 Hospital Universitário Clementino Fraga Filho, Universidade Federal do Rio de Janeiro, Rio de Janeiro, Brazil; 4 Universidade Federal de Santa Catarina, Florianópolis, Brazil; 5 Centro de Hemoterapia e Hematologia do Pará, Belém, Brazil; 6 Laboratório Central de Saúde Pública do Amazonas, Manaus, Brazil; 7 Centro de Ciências Biológicas e da Saúde, Universidade Federal de Mato Grosso do Sul, Campo Grande, Brazil; 8 Laboratório Central de Saúde Pública de PernambucoDr. Milton Bezerra Sobral, Recife, Brazil; 9 Instituto de Patologia Tropical e Saúde Pública, Departamento de Microbiologia, Universidade Federal de Goiás, Goiânia, Brazil

## Abstract

**Background:**

Hepatitis B virus (HBV) isolates have been classified in eight genotypes, A to H, which exhibit distinct geographical distributions. Genotypes A, D and F are predominant in Brazil, a country formed by a miscegenated population, where the proportion of individuals from Caucasian, Amerindian and African origins varies by region. Genotype F, which is the most divergent, is considered indigenous to the Americas. A systematic molecular characterization of HBV isolates from different parts of the world would be invaluable in establishing HBV evolutionary origins and dispersion patterns. A large-scale study is needed to map the region-by-region distribution of the HBV genotypes in Brazil.

**Results:**

Genotyping by PCR-RFLP of 303 HBV isolates from HBsAg-positive blood donors showed that at least two of the three genotypes, A, D, and F, co-circulate in each of the five geographic regions of Brazil. No other genotypes were identified. Overall, genotype A was most prevalent (48.5%), and most of these isolates were classified as subgenotype A1 (138/153; 90.2%). Genotype D was the most common genotype in the South (84.2%) and Central (47.6%) regions. The prevalence of genotype F was low (13%) countrywide. Nucleotide sequencing of the S gene and a phylogenetic analysis of 32 HBV genotype F isolates showed that a great majority (28/32; 87.5%) belonged to subgenotype F2, cluster II. The deduced serotype of 31 of 32 F isolates was *adw4*. The remaining isolate showed a leucine-to-isoleucine substitution at position 127.

**Conclusion:**

The presence of genotypes A, D and F, and the absence of other genotypes in a large cohort of HBV infected individuals may reflect the ethnic origins of the Brazilian population. The high prevalence of isolates from subgenotype A1 (of African origin) indicates that the African influx during the colonial slavery period had a major impact on the circulation of HBV genotype A currently found in Brazil. Although most genotype F isolates belonged to cluster II, the presence of some isolates belonging to clusters I (subgroup Ib) and IV suggests the existence of two or more founder viral populations of genotype F in Brazil.

## Background

Hepatitis B virus (HBV) is the prototype of the *Hepadnaviridae *family characterized by DNA viruses with tropism to hepatic cells. HBV is an etiologic agent of human liver diseases, including acute and chronic hepatitis, cirrhosis, and hepatocellular carcinoma. As such, it constitutes a significant public health problem, estimated to chronically infect more than 350 million people worldwide (reviewed in [1]).

The HBV genome is a partially double-stranded circular DNA molecule approximately 3.2 kb in length. The viral proteins are encoded by four partially overlapping open reading frames (ORFs), which allow the virus to produce 50% more proteins than would be predicted by the small genome size. HBVs also have an unusual replication strategy. Because they first synthesize RNA intermediates from which DNA is synthesized by reverse transcription, the HBV DNA viruses have substitution rates more than 10-fold higher than other DNA viruses.

On the basis of genetic differences, HBV have been classified into eight genomic groups (A-H). Entire genome sequences within each group diverge from other groups by more than 8%. These eight groups have distinct geographical distributions [2,3]. Genotype A is distributed globally and is the main genotype found in Europe, North America, Africa and India. Genotypes B and C are predominant in East and Southeast Asia, and Australia. Genotype D is mainly found in the Middle East and Mediterranean countries but has been reported globally, whereas genotype E seems to be predominant in West Africa. Genotype G has been characterized in samples from USA, Mexico and France, and genotypes F and H are found exclusively in Central and South America (reviewed in [4,5]).

Some genotypes have been further subdivided into subgroups (subgenotypes) with distinct geographic origins. The HBV genotype A strain is now divided into three genetic clusters. Subgenotype A1 represents isolates with an African-Asian origin, whereas subgenotype A2 includes isolates with European-North American origin [6]. Recently, a new subgenotype, designated A3, was described in Cameroon [7]. Genotypes B [3,8-10], and C [3,11,12] have each been subdivided into four subgenotypes, and genotype D [3,13,14] has been subdivided into five subgenotypes. Genotype F, which constitutes the most divergent group [15], is indigenous to the Americas and is the major circulating genotype in Argentina [16-18], Venezuela [19,20], the Peruvian Amazon Basin [21], Northern Brazil [22] and Central American countries, including Costa Rica, El Salvador and Nicaragua [23]. Recently, two subgenotypes, F1 and F2, have been proposed for this genotype, each characterized by a specific amino acid residue, Leu^45 ^and Thr^45^, respectively, in the small (S) gene product [24]. A subdivision of isolates from genotype F into five clusters (Ia, Ib, II, III and IV) has also been proposed by Mbayed *et al*. on the basis of comparisons between S gene sequences [25]. Clusters Ia and Ib, associated with subgenotype F1, are representative of strains found mainly in Central America and Argentina, respectively. Subgenotype F2 includes clusters II (Nicaragua, Venezuela, and Brazil), III (Panama, Venezuela, and Colombia) and IV (Argentina and Bolivia) (reviewed in [4]).

Brazil is a federation of 26 states and one federal district occupying approximately 8,500,000 km^2 ^divided broadly into five geographic regions: North, Northeast, Central-West, Southeast and South. Early studies have shown variability in HBV prevalence in different Brazilian regions. The Amazon basin, corresponding to the North region and the northern portions of the Central-West region, is characterized by a rate of endemic HBV infection, in contrast to the low prevalence found in southern regions of the country [22,26-28]. Brazil, a country with a highly miscegenated population, exhibits an HBV genotype circulation pattern that is distinct from the distribution found in other Latin American countries, with genotypes A, D and F being the most prevalent among HBV carriers [29-31].

In this study, we performed a molecular characterization of HBV strains derived from HBsAg-positive blood donors living in different Brazilian regions. S region nucleotide sequencing and phylogenetic analysis were performed to determine the relationships between HBV genotype F isolates from Brazil and from other American countries.

## Results

### Distribution of HBV genotypes in Brazil

**Figure 1:**
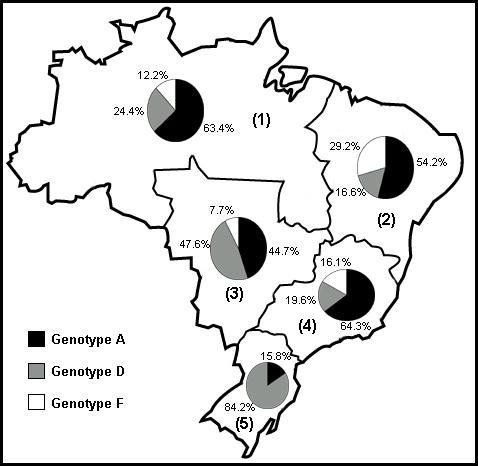
**Distribution of HBV genotypes in different Brazilian regions**. Map representing HBV genotypic distribution in all five Brazilian regions (1 – North region; 2 – Northeast region; 3 – Central-West region; 4 – Southeast region; 5 – South region).

Genotyping by PCR-RFLP of 303 HBV isolates from all five Brazilian geographic regions demonstrated that genotypes A, D and F were present at different frequencies; no other genotypes were identified in the samples analyzed. Genotype A was the most prevalent in the North (52/82; 63.4%), Northeast (13/24; 54.2%) and Southeast (36/56; 64.3%) regions. Genotype D was the most prevalent in South (32/38; 84.2%), whereas genotype F isolates were absent in this region (Figure 1). In the center of the country, represented by the Central-West region, there was a balanced distribution of genotypes A (46/103; 44.7%) and D (49/103; 47.6%). Countrywide, genotype A was the most common (48.5%), followed by genotype D (38.5%) and genotype F (13%).

As shown in a previous work [31], the PCR-RFLP genotyping method divided each genotype in different RFLP patterns. In the current study, a total of thirteen RFLP patterns were observed (Table 1). Genotype A isolates were subdivided into four patterns, AI-AIV, the great majority of which were classified as AI or AII (138/153; 90.2%). Among the five RFLP patterns found for genotype D (DI-DV), patterns DI and DII were the most prevalent (92/116; 79.3%). For genotype F, FI was the most frequent pattern (20/34; 58.8%) of the four patterns found (FI-FIII, FV).

**Table 1:** Distribution of RFLP patterns in HBV isolates of HBsAg-positive blood donors living in different Brazilian geographic regions.

	**RFLP patterns**													
		
**Brazilian regions**	**Genotype A**				**Genotype D**					**Genotype F**				**TOTAL**
				
	**AI**	**AII**	**AIII**	**AIV**	**DI**	**DII**	**DIII**	**DIV**	**DV**	**FI**	**FII**	**FIII**	**FV**	
South	3	1	2	-	15	13	-	3	1	-	-	-	-	38
Southeast	21	8	6	1	4	7	-	-	-	3	3	2	1	56
Central-West	34	8	3	1	18	15	2	14	-	6	1	-	1	103
Northeast	5	8	-	-	4	-	-	-	-	5	-	2	-	24
North	31	19	2	-	12	4	1	3	-	6	-	3	1	82

**TOTAL**	94	44	13	2	53	39	3	20	1	20	4	7	3	**303**

### Phylogenetic analysis of the small (S) gene and amino acid replacements

The results of PCR-FRLP analysis were confirmed by direct nucleotide sequencing of HBV S gene PCR products from 62 of 303 samples (20.5%), including randomly selected samples from genotype A (22 samples) and genotype D (13 samples), and 27 samples from genotype F. No discrepancies were found between the two methods. Among genotype A samples, 15/22 (68.2%) were classified as subgenotype A1 and 7/22 (31.8%) as subgenotype A2. A phylogenetic analysis of genotype D samples assigned 10/13 (76.9%) to subgenotype D3 and 3/13 (23.1%) to subgenotype D2. The deduced amino acid sequence of the S gene product and an analysis of antigenic determinant *a *residues 122 and 127 confirmed the expected serotype pattern of all but one genotyped sample. All 22 HBV genotype A samples were classified as serotype *adw2*. Nine genotype D samples were classified as serotypes *ayw2 *and three were identified as *ayw3*. Of the 32 HBV genotype F samples, 31 were *adw4*, and one, 052-N, had an unusual substitution of isoleucine for leucine at residue 127. Three HBV genotype A isolates (358-S; 312-SE; 161-CW) were shown to harbor tyrosine to cysteine substitutions in determinant *a *residue 100 (Y100C), and the T118V-A128V double mutant was found in three genotype D isolates (Table 2). The partial amino acid sequence of HBV polymerase showed amino acid substitutions related to the lamivudine-resistance phenotype in two isolates derived from this group of HBsAg positive blood donors. One (209-CW) displayed the double rtL180M-rtM204V lamivudine-resistance mutation and the other (043-N) showed an additional rtV173L mutation, which is also associated with lamivudine resistance [32] (Table 2).

**Table 2:** S protein amino acid replacements and lamivudine-resistant rt domain polymerase mutations found in blood donors.

**Sample**	**Genotype**	**Serotype**	**Determinant *a *replacements**	Lamivudine resistance mutation (P gene)*
358-S	A	*adw2*	Y100C	-
312-SE	A	*adw2*	Y100C	-
161-CW	A	*adw2*	Y100C	-
043-N	A	*adw2*	-	rtV173L, rtL180M, rtM204V
020-N	D	*ayw3*	T118V, A128V	-
328-S	D	*ayw3*	T118V, A128V	-
344-S	D	*ayw3*	T118V, A128V	-
209-CW	F	*adw4*	-	rtL180M, rtM204I
052-N	F	*adw?*	L127I	-

*Deduced amino acid sequence of HBV polymerase based on the shifted reading frame of the sequenced S gene (P gene: aa rt 9–236).

### Molecular characterization of genotype F isolates

**Figure 2:**
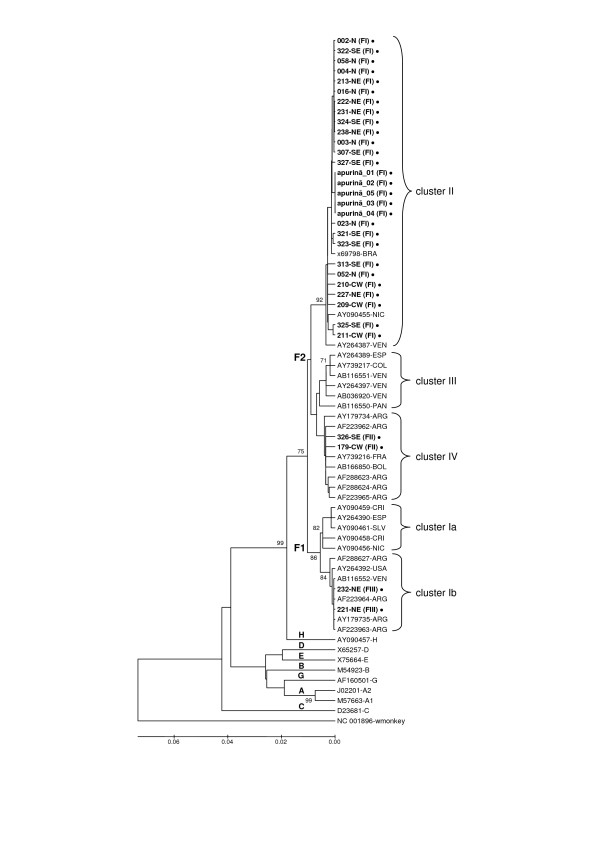
**Phylogenetic-tree representing HBV genotype F isolates**. Brazilian sequences determined in this study are represented in bold (•), designated by the corresponding region of the sample (N: North region; NE: Northeast region; CW: Central-West region; SE: Southeast region) with RFLP pattern in brackets. International sequences are designated with their accession number followed by their countries of origin.

A phylogenetic analysis of the S gene sequences from 32 HBV genotype F isolates (27 from blood donors and 5 previously characterized serum samples from the native Amerindian Apurinã tribe) in a set with 27 international HBV genotype F sequences (GenBank), classified genotype F isolates into five different subgroups (Figure 2). Using the topology of the phylogenetic tree, we were able to identify the subgenotypes F1 and F2 (Figure 2). The vast majority of Brazilian genotype F strains determined in the present study (28/32; 93.75%) were classified as subgenotype F2, whereas only two samples (2/32; 6.25%) were grouped into subgenotype F1. An amino acid analysis supported this finding, showing that all samples grouped into the F2 subgenotype by the phylogenetic analysis contained a threonine at position 45 of the S gene product, whereas the two subgenotype F1 samples had a leucine at this position (data not shown). The tree topology also allowed us to identify the five genotype F clusters (Figure 2). Twenty-eight of 32 samples analyzed (87.5%) were classified into cluster II and 2 samples (6.25%) were assigned to cluster IV; the two subgenotype F1 samples were related to cluster Ib. Correlating the RFLP patterns and the phylogenetic clusters of isolates belonging to genotype F for all Brazilian samples, we found that samples characterized as RFLP pattern FI were grouped in cluster II, whereas those classified as FII and FIII on the basis of RFLP patterns were related to isolates from cluster IV and Ib, respectively.

## Discussion

The previously described co-circulation of HBV genotypes A, D and F in Brazil [31,33-39] was confirmed in the present study, which also found that no other HBV genotypes were present in a sample of 303 blood donors. The identification of genotype prevalence patterns in Brazil that are distinct from worldwide geographic distribution patterns could be a reflection of the intensely migratory and highly miscegenated character of the Brazilian population. Genotypes B and C, previously identified in a few Brazilian individuals, all of Asian origin [36,40], have not been detected in the present study. The absence of genotypes B and C may be explained by a combination of a limited sample size and an unbalanced distribution of individuals from Asian origin throughout the Brazilian territory (reflecting the tendency of Asian immigrants to concentrate in certain restricted geographic areas).

The distribution of HBV genotypes A and D in Brazil seemed to follow a gradient from northern to southern regions (Figure 1). North, Northeast and Southeast regions showed a higher prevalence of genotype A, the most common genotype in Brazil. The high rate of genotype D isolates in the South region could be related to the influx of immigrants from Central Europe (especially Germany and Italy) that occurred in that region at the beginning of the 20^th ^century. The balanced distribution of genotypes A and D in the Central-West region could be explained by the delayed occupation of that area by population migration flows from South, Southeast and Northeast regions.

HBV genotype A had been initially divided into two genetic subgroups, A1 and A2, on the basis of sequence divergence [6,7]. A previously described PCR-RFLP genotyping method has classified HBV isolates into more than 20 patterns [31]. Sequences showing patterns AI, AII and AVI clustered in subgroup A1, whereas those showing patterns AIII and AV were found exclusively in subgroup A2. In the present study, the RFLP and sequence analyses of genotype A isolates circulating in Brazil showed a high prevalence of isolates from subgroup A1 (90.2%, Table 1), corroborating previous results and indicating that the HBV genotype A circulating in Latin America is not exclusively associated with the European influx, as once thought (reviewed in [4]). On the contrary, the massive African forced migration during the colonial times (from the 16^th ^to 19^th ^century) was a major contributor to the HBV circulation currently found in Brazil. The absence of genotype E, which circulates in sub-Saharan Africa where substantial slave traffic occurred, could be explained by the relatively recent emergence of this genotype as a human pathogen, dating from the mid to late 19^th ^century [41] when the slave trade had already been abolished.

A large-scale study on the geographic distribution of HBsAg subtypes in Brazil, published in the late 1980s, reported a high incidence of *adw2 *(associated with genotype A) in all regions, except the South region, where subtypes *ayw2 *and *ayw3 *(both associated with genotype D) were the most prevalent [22]. These results are in accord with our current results. However, other findings of this previous study, including the reported absence of subtypes other than *adw2 *in Northeast region, a vast majority of the *adw2 *subtype in the Central-West region, and the high prevalence of subtype *adw4 *(genotype F) in the North region, are not corroborated by our study. These discrepancies may be explained by differences in methodology: Because the former study classified HBV isolates on the basis of differences in antigenic determinants [22], which usually requires high titers of HBsAg for subtype determination, it may have mischaracterized low titer samples that are readily classified using genotypic analyses. Alternatively, divergent conclusions could arise due to differences in sampling that affect the population under study and lead to a genotype prevalence bias with an underestimation of genotype F. For example, the current study analyzed serum samples from blood banks located in state capitals of Brazil, a sampling strategy that would tend to under-represent certain groups, such as Amerindians, that are not often voluntary blood donors. It is also possible that a given genotype induces more symptomatic infection, leading to a reduction in its prevalence in the potential blood donor population. Further studies comparing clinical outcomes in patients infected with genotypes A, D and F will be required to address this question.

In general, HBV genotype F, which is native to the Amerindians, showed a low prevalence in Brazil (13%). This was true even in the North region where the native Amerindian population comprises a larger fraction of the total population. This result supports the findings of Moraes *et al*. [33] and contrasts with the high prevalence of genotype F described in other South American countries, including Venezuela [19,20] and Argentina [16,18]. These data indicate that, in Brazil, the native Amerindian population has made a minor contribution to the population as a whole, a fact supported by an analysis of mitochondrial DNA [42,43].

As a starting point for establishing a molecular characterization of Brazilian HBV genotype F isolates, we determined S gene sequences. When combined with a phylogenetic analysis, nucleotide sequences of individual genes, in particular the S gene, can reveal relationships that help to elucidate the molecular epidemiology of HBV [4,25,44,45]. The topology of the HBV genotype F phylogenetic tree showed that the great majority of Brazilian strains characterized in the present study could be classified as subgenotype F2, cluster II. Two previous studies have described one Brazilian sequence belonging to cluster II [17,25]. In the present study, HBV genotype F isolates with sequences closely related to those grouped in clusters IV and Ib were found, suggesting that Brazilian genotype F strains may not be derived from one another. Assuming that the five samples previously characterized as genotype F from the Apurinã tribe (apurina_01–05) represent the native original HBV of the Brazilian Indians, and further assuming that the great majority of Brazilian genotype F sequences determined in this study was closely related to them, it is reasonable to infer that HBV genotype F circulating in Brazil is mainly derived from the native aboriginal populations. However, the differences observed between the two sequences grouped in cluster Ib (232-NE; 221-NE) and the other genotype F sequences could indicate that two or three viral populations with distinct origin circulate in Brazil. Moreover, the existence of this well-defined cluster as the representative Brazilian isolate would indicate that HBV genotype F arose from a common ancestor, but evolved separately after becoming isolated in different regions of the Americas.

Genotyping using PCR-RFLP analysis and nucleotide sequencing gave the same result for all 62 samples tested, indicating that our PCR-RFLP genotyping method is appropriate for large-scale studies. Similar to our previous report on genotype A isolates [31], our results here demonstrate an interesting correlation between the RFLP patterns identified and the cluster analysis of HBV genotype F isolates. All FI RFLP-pattern isolates subjected to nucleotide sequencing were found to group into genotype F cluster II, whereas the two isolates grouped in cluster IV (326-SE; 179-CW) and the two isolates grouped in cluster Ib (232-NE; 221-NE) were classified as RFLP pattern FII and FIII, respectively. Although more extensive sampling will be necessary to confirm this relationship, it is possible that RFLP patterns could be used to establish to which cluster the HBV genotype F isolate is related.

The identification of a novel isoleucine substitution at residue 127 in one HBV genotype F isolate (052-N) classified by serotype using amino acid analysis reinforces the need for further investigation of genotype F strains. The deduced amino acid sequence of the S gene product also revealed three isolates bearing the determinant *a *substitution Y100C, which is associated with the HBsAg-negative/anti-HBc-positive phenotype in blood donors from Venezuela [46] and has been found in an HBsAg-positive Brazilian patient [47]. Further studies will be required to determine whether this mutation is truly related to a reduction in HBsAg detection. The T118V-A128V double mutation was found in three genotype D/*ayw3 *(subgenotype D2) isolates. These substitutions, which have been found in other subgenotype D2 isolates [3], have recently been identified in Brazil (unpublished data), Spain, Sweden and Poland [48].

The deduced amino acid sequence of HBV polymerase showed that two isolates had substitutions in residues associated with lamivudine resistance. The frequent lamivudine-resistance rtL180M-rtM204V double mutation was detected in two isolates (043-N and 209-CW), while a third isolate (043-N) displayed a rare rtV173L-rtL180M-rtM204V triple mutation (Table 2). This triple mutant is known to exhibit reduced *in vitro *affinity for anti-HBs antibodies, similar to the hepatitis B vaccine escape mutant G145R [32,49]. These findings were unexpected given that the samples analyzed in the present study were derived from blood donors, a group of individuals not undergoing lamivudine therapy. However, lamivudine-resistant isolates would eventually be expected to circulate in the environment as a result of contact with chronic hepatitis B patients undergoing lamivudine antiviral therapy, allowing healthy individuals, including blood donors, to become exposed to a primary infection with an HBV mutant strain. The presence of lamivudine-resistant isolates could also be related to previously reports of HBV isolates with a YMDD motif mutation in chronic hepatitis B patients not treated with lamivudine [50,51].

The evolutionary origins of HBV remain ill defined. Although some early studies have suggested a New World origin for this virus, the data are ambiguous and specific inferences should be drawn with caution [52,53]. The diversification of HBV genotypes is poorly understood, but estimates based on phylogenetic divergence rates have calculated that HBV genotypes diverged less than 6000 years ago (reviewed in [54]). The origin of genotype F is particularly obscure, and may represent the first split from the human hepadnaviral ancestor (reviewed in [12]). As many authors have noted, a refined analysis of the evolutionary history of HBV will require more extensive sampling of HBV genomes.

## Conclusion

The distribution of HBV genotypes A and D identified in the present study followed an apparent gradient from northern to southern regions of Brazil, possibly reflecting the influence of early populations and settlement patterns during the process of occupying the country. The high prevalence of isolates from subgroup A1 (90.2%) may denote a major contribution of forced African influx during the colonial slavery period to the current HBV genotype A circulation in Brazil.

In contrast to other Latin American countries, HBV genotype F showed a low prevalence in Brazil (13%), even in the North region where the native aboriginal population has a greater influence. The topology of the phylogenetic tree for HBV genotype F strains allowed the classification of most strains into subgenotype F2 within cluster II. The existence of this well-defined cluster as the representative Brazilian isolate would indicate that HBV genotype F arose from a common ancestor, but evolved separately after becoming isolated in different regions of the Americas. The presence of some isolates of clusters IV and I (subgroup Ib) argues for the existence of two or more founder viral populations of genotype F in Brazil. Because the diversification of HBV genotypes remains poorly understood, particularly that of genotype F, more extensive sampling of HBV genomes would be helpful in unraveling the complex evolutionary history of HBV.

## Methods

### Serum samples

A total of 303 serum samples from HBsAg-positive blood donors were collected between 2003 and 2004 at blood banks located in state capitals cities of Brazil representing different Brazilian regions. Thirty-eight samples were from the Santa Catarina State (South region); 56 samples were from Rio de Janeiro State (Southeastern region); 103 samples were from the three states that compose Central-West region: Goiás (18), Mato Grosso (55), and Mato Grosso do Sul (30); 24 samples were from Pernambuco State (Northeast region); and 82 samples were from the North region (35 from Pará, 34 from Amazonas, and 13 from Amapá). In addition, five HBsAg-positive serum samples from the native Amerindian tribe Apurinã (Amazonas, Brazil), previously characterized as genotype F by PCR-RFLP, were included in the study.

### DNA extraction, amplification and RFLP analysis

HBV DNA was phenol/chloroform extracted from 250 μL of serum after treatment with 0.5 mg/mL of proteinase K for 4 h at 37°C in the presence of 0.2 M NaCl and 0.25% SDS, as previously described [55]. After precipitation with ethanol, the pellet was dried and resuspended in 30 μL of distilled water. The pre-S/S genome region was amplified by semi-nested PCR. The first round contained one sense primer (C1, 5'-CTGTGGAGTTACTCTCGTTTTTGC-3', nt positions 1935–1958) and two antisense primers (S2, 5'-GGGTTTAAATGTATACCCAAAGA-3', 819–841, and S22, 5'-GTATTTAAATGGATACCCACAGA-3', 819–841) located at the same position on the genome to facilitate the amplification of all HBV genotypes [33]. After an initial denaturation step (3 min at 94°C), DNA was amplified using 35 cycles of 94°C for 40 s, 55°C for 1 min, and 72°C for 2 min 30 s, followed by a final elongation step (7 min at 72°C). The first round of amplification was performed with 1 μL of DNA and one unit of Taq DNA polymerase (Invitrogen, San Diego, CA) in a final volume of 25 μL. The second round of amplification was performed in a final volume of 50 μL, using 1 μL of the first round PCR product, sense primer PS1 (5'-CCATATTCTTGGGAACAAGA-3', 2826–2845) and antisense primers S2 and S22, under the following conditions: 30 cycles of 95°C for 30 s, 52°C for 40 s, and 72°C for 2 min, followed by a final elongation step (7 min at 72°C). Ten microliters of amplification product (about 1,200 bp in length) was loaded on 1% agarose gels, electrophoresed, stained with ethidium bromide and visualized under UV light. Genotyping by RFLP analysis was performed as previously described [31] using 10 μL of PCR products digested separately with two units of *Bam*HI, *Eco*RI and *Stu*I restriction endonucleases (Roche Molecular Biochemicals, Mannheim, Germany) at 37°C for 2 h. Digestion products were analyzed by electrophoresis in 2% agarose gel.

### Nucleotide sequencing and phylogenetic analysis

The HBV S gene from 62 RFLP-genotyped samples was sequenced to verify the accuracy of our RFLP-based genotyping method. Twenty-two samples characterized as genotype A and 13 samples as genotype D by the RFLP-based method were randomly chosen for nucleotide sequencing. Twenty-seven samples determined as genotype F and 5 samples from native Amerindian tribe Apurinã (Amazonas, Brazil) previously characterized as genotype F were also sequenced. The S gene was first amplified by PCR using a mixture of one sense primer (S1, 5'-CTTCTCGAGGACTGGGGACC-3', nt positions 124–143) and two antisense primers (S2, S22, described above) followed by purification of PCR products using QIAquick Gel Extraction Kit (QIAGEN, Valencia, CA). Purified PCR products were prepared for sequencing using a Big Dye Terminator 3.1 Cycle Sequencing Kit (Applied Biosystems, Foster City, CA) with external primers S1 and S2 or S22, internal sense primer S4 (5'-TGCTGCTATGCCTCATCTTCT-3', nt 416–436) and antisense S7 (5'-TGAGCCAGGAGAAACGGGCT-3', nt 676–656). The sequence was determined by separation and analysis of extension products using an automated ABI 3730 DNA Analyzer (Applied Biosystems, Foster City, CA).

Raw HBV S gene sequence data were processed using programs from the Genetic Computer Group (GCG) package (University of Wisconsin, Madison, WI). Phylogenetic analysis was performed by comparing 681 bp sequences from the HBV S gene determined in this study with HBV sequences available at GenBank. Sequences were aligned using the Clustal W program [56] and the phylogenetic-tree analysis was performed using the neighbor-joining method (bootstrap resampling test with 1,000 replicates) in MEGA version 3.0 software [57]. All sequences determined in this study have been deposited in the GenBank database [GenBank: EF690470 – EF690536].

## Authors' contributions

FCAM carried out the molecular biology experiments and wrote the manuscript. FJDS, LCN, CAVN, HSMC, HCFF, JCPS, HAV, ARCMC, MMMM and RMBM were responsible for HBsAg evaluation and the epidemiological data of HBsAg positive blood donors. SAG conceived the study, participated in its design and coordination, and corrected the final version of the manuscript. All authors read and approved the final manuscript.
